# The Role of Endocrine Stress Systems and Sex Hormones in the Enhancing Effects of Stress on Mental Rotation Capabilities

**DOI:** 10.3390/brainsci10110791

**Published:** 2020-10-29

**Authors:** Ami Cohen, Or Chen Zemel, Raul Colodner, Randa Abu-Shkara, Refaat Masalha, Lila Mahagna, Efrat Barel

**Affiliations:** 1 Department of Psychology, The Max Stern Yezreel Valley College, Afula 1855701, Israel; 2 Department of Behavioral Sciences, The Max Stern Academic College of Emek Yezreel, Afula 1855701, Israel; orchen.b@gmail.com (O.C.Z.); efratb@yvc.ac.il (E.B.); 3 Laboratory Medicine Department, Emek Medical Center, Afula 1855701, Israel; colodner_ra@clalit.org.il (R.C.); randaa85@gmail.com (R.A.-S.); masalha_re@clalit.org.il (R.M.); lila_ma@clalit.org.il (L.M.)

**Keywords:** Trier social stress test, alpha-amylase, cortisol, sex hormones, mental rotation, visuospatial

## Abstract

The possible effects of stress and neurobiological stress mechanisms on visuospatial abilities remain largely unknown. In the current study, we examined the combined effect of sex hormones and both the hypothalamic–pituitary–adrenal axis (HPA-A) and the sympathetic nervous system (SNS) on stress-induced changes in visuospatial performance. A total of 107 participants completed a mental rotation task and were subsequently exposed to either to the Trier social stress test (TSST) or to a control condition before completing the mental rotation task again. HPA-A and SNS reactivity of the participants were evaluated by measuring salivary alpha amylase (sAA; an SNS activation marker) and cortisol in four saliva samples. Pre-stress levels of sex hormones (progesterone, estradiol, and testosterone) were also measured. The TSST enhanced mental rotation performance, and this enhancement was negatively correlated with baseline estradiol levels and positively correlated with the level of cortisol reactivity among men. In addition, controlling for baseline levels of testosterone, estradiol, and progesterone diminished this effect of stress. These results imply that the stress-induced facilitation of mental rotation performance is modulated by baseline sex hormones and provide preliminary support to the notion that a complex interaction between sex hormones and neuroendocrine stress mechanisms mediates the influence of stress on visuospatial performance.

## 1. Introduction

It is well established that exposure to stress can affect various cognitive functions [1,2,3]. However, only few studies have addressed the possible effects of stress and neurobiological stress mechanisms on visuospatial abilities. These studies yielded mixed results, with psychosocial stress improving spatial navigation in one study [4] but impairing it in another [5]. Brain regions implicated in mental rotation, a major subtype of visuospatial abilities, such as the anterior hippocampus [6] and the inferior parietal cortex [7], contain cortisol receptors and/or are functionally influenced by cortisol [8,9]. However, the involvement of the stress systems (hypothalamic–pituitary–adrenal axis (HPA-A) and the sympathetic nervous system (SNS) in visuospatial performance is unclear. To the best of our knowledge, the role of SNS activation in the effects of stress on visuospatial performance has not been studied to date, and studies on the possible role of HPA-A activation have produced mixed results. Specifically, under nonstress conditions, free cortisol levels and visuospatial performance of young adults have been shown to be positively associated in one study [10] but showed no correlation in another [11]. Furthermore, although cortisol administration was shown to impair spatial performance under nonstress conditions [12], cortisol levels in this study were high in relation to the physiological range. Thus, the role of HPA-A activation and SNS activation in the effects of stress on visuospatial performance remains uncertain. Given that the neurobiological mechanisms underlying reproduction and stress response are interlinked [13], sex hormones may modulate the effects of stress in general, and on visuospatial capabilities in particular. Indeed, a negative correlation has been demonstrated between post-stress testosterone levels and cortisol reactivity [14]. Progesterone and estradiol also appear to modulate the physiological stress response, though in a sex-dependent manner. Specifically, the association between basal progesterone levels and cortisol reactivity to stressors was shown to be negative among men [14] but positive among women at the follicular stage of their menstrual cycle [15]. Moreover, delivery of estradiol or progesterone enhanced the SNS and HPA-A stress responses among men but attenuated them in menopausal women [16,17,18]. Moreover, recent findings suggest that the effects of stress on cognitive functions, such as declarative memory, may be modulated by interrelationships between sex hormones and neuroendocrine stress systems (SNS and HPA-A) [19].

Thus, the current study aimed at examining the interaction between the stress systems (SNS and HPA-A) and baseline reproductive hormones in mediating the effects of psychosocial stress on mental rotation. HPA activation and SNS activation were evaluated noninvasively by measuring salivary cortisol and salivary alpha-amylase (sAA; an SNS activation marker). In previous studies, estradiol administration to elderly women increased cortisol stress reactivity and mental rotation performance [20], and psychosocial stress improved spatial navigation among women but not among men [21]. Therefore, we hypothesized that stress would enhance mental rotation performance and that this enhancement would be modulated by the levels of cortisol and sAA as well as sex hormones in general and estradiol in particular.

## 2. Materials and Methods

### 2.1. Participants

The study sample included 107 young men (*N* = 37) and women (*N* = 70) with a mean age of 24.47 years (SD = 2.63) and a mean BMI of 23.24 (SD = 3.21). Of the female participants, 34 were taking oral contraceptives (oral contraceptives group; OC). The other 36 were not using oral contraceptives and were at the mid-luteal phase (day 21) of their menstrual cycle at the time of the study (luteal phase group; LP). Participants were recruited from among college students using advertisements and received monetary compensation ($25) for their participation. After signing an informed consent form, the volunteers completed a brief demographic questionnaire. The participants reported that they have no serious medical, gynecological, or hormonal problems and no psychopathologies that may affect hormonal regulation (e.g., depression), ADHD, nor learning disabilities, and were nonsmokers. In addition, women in the OC group reported using contraceptive pills containing 25 mg of estradiol (ethinylestradiol) and 75 mg of progestin (gestodene). These doses are considered moderate and are commonly prescribed. The women included in the LP group did not use oral contraceptives for at least six months prior to the study, had a regular menstrual cycle, and were not pregnant or lactating. These participants were monitored for at least 3 months prior to the study to verify the regularity of their cycle and reported for testing to the research laboratory on the twenty-first day of their cycle using the day of onset of the last menstruation as a reference point. Participants had to be awake for at least 1 h before testing to enable control of circadian fluctuations in cortisol. The study was approved by the Institutional Review Board of the college. The study was approved by the Ethics Review Board of Max Stern Yezreel Valley College (approval number: 2020-15 YVC EMEK).

### 2.2. Experimental Procedure

**Figure 1:**
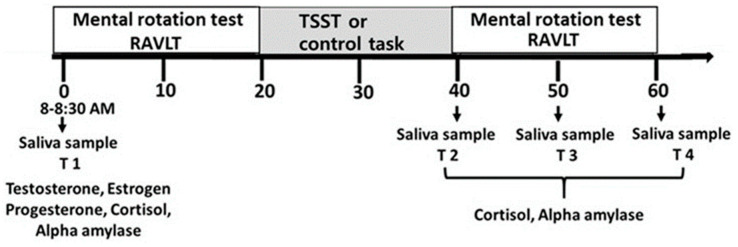
Study design.

This study included examination of the effects of stress on both verbal memory and mental rotation performance. An elaborated description of the procedure of this study has been previously reported in a paper describing the findings regarding the effects of TSST on participant’s verbal memory [19]. The experimental sessions took place in the laboratory of the YVC Psychology Department between 8:00–10:00 a.m., a time period during which testosterone levels are at their peak [22,23]. All participants were tested more than an hour after awakening to avoid possible confoundment between cortisol rise due to stress induction and rise due to the cortisol awakening response [24]. Participants from each hormonal status group (men, OC, LP) were randomly assigned to one of the two experimental groups, stress and control, and completed three stages (see Figure 1): (1) completion of the mental rotation test and the Rey auditory verbal learning test (RAVLT) (20 min total, 10 min for each test); (2) the Trier social stress test procedure or the control condition (20 min); and (3) completion of the mental rotation test and the RAVLT (20 min total, 10 min for each test). The stimuli included in the tests and their order differed in Stages 1 and 3. In addition, the order of the mental rotation test and the RAVLT was counterbalanced. The participants provided saliva samples at four assessment points: T1 (baseline: 8:00–8:30 a.m.), T2 (immediately following the TSST/control), T3 (T2 + 10 min), and T4 (T3 + 10 min). For the T1 sample, participants provided 5 mL of saliva, which was used to evaluate levels of testosterone, estradiol, and progesterone as well as baseline levels of cortisol and sAA. For the other samples, participants provided 2 mL of saliva, which was used to measure levels of reactive cortisol and sAA.

The experimental session included the following consecutive stages: (A) the mental rotation task and the Rey auditory verbal learning test (RAVLT) (not reported here); (B) the Trier social stress test procedure or the control procedure; and (C) the mental rotation task and the RAVLT (not reported here). Participants provided saliva samples at the four assessment points indicated as T1–T4. Participants provided 5 mL of saliva at T1 and 2 mL of saliva for T2–T4.

### 2.3. Saliva Sampling Procedure and Biochemical Analysis

The participants were instructed to refrain from eating and drinking (aside for water) for at least 1 h prior to the experimental session. Before each saliva sampling, participants chewed on a piece of parafilm for several seconds to increase saliva secretion. They then provided a sample of saliva in a SaliCap sampling vial. The duration of time it took the participants to produce the needed amount of saliva varied between 10 to 120 s. Notably, chewing may affect the relative amount of alpha-amylase in the saliva [25]. However, this factor was kept constant across the study groups. Saliva samples were stored at −20 °C immediately after collection. For each biochemical analyte, tests were performed using commercial CE-IVD-approved ELISA kits: 17 Beta Estradiol Saliva ELISA (mean intra-assay CV% = 4.8, mean inter-assay CV% = 3.4, assay sensitivity = 0.4 pg/mL), Cortisol Saliva ELISA (mean intra-assay CV% = 4.8, mean inter-assay CV% = 8.1, assay sensitivity = 0.005 µg/dL), Testosterone Saliva ELISA (mean intra-assay CV% = 9.1, mean inter-assay CV% = 5.7, assay sensitivity = 2.0 pg/mL), Progesterone Saliva ELISA (mean intra-assay CV% = 5.2, mean inter-assay CV% = 7.0, assay sensitivity = 3.1 pg/mL), Alpha-Amylase Saliva ELISA (mean intra-assay CV% = 4.6, mean inter-assay CV% = 6.2, assay sensitivity = 3.6 U/mL), all from IBL International GMBH, Hamburg, Germany). All tests were conducted in an SQII ELISA processor (AESKU Systems, Wendelsheim, Germany). A calibration curve using standard duplicates was performed for each analyte in every run. The performance of all kits were validated in our laboratory according to good laboratory practice (GLP) guidelines, complying with ISO 9001 certification and JCI accreditation standards.

### 2.4. Trier Social Stress Test and the Nonstress Control Condition

We subjected the participants to the TSST [26]. This task included 5 min of free speech (job interview) and 5 min of a mental arithmetic task, both conducted in front of a video camera and a committee comprising a man and a woman. The participant remained standing at a distance of 1.5 m from the committee. At the beginning of the procedure, the committee members gave instructions to the participants regarding the task at hand and told them that their performance would be recorded for subsequent behavioral analysis. The participants were then taken to an empty room in which they had 10 min to prepare for the task. After this, the participants entered the committee room where they performed stress task. In total, the procedure, including the preparation phase, took approximately 20 min.

The control condition was similar to the stress condition except that the stressful task was replaced by a control task [27]. The procedure consisted of a 10 min phase during which each participant was required to read the entry “England” in Wikipedia silently, followed by 5 min of reading the entry “transport in Israel” out loud and another 5 min of counting out loud. During the entire 20 min of the task, the participant was alone in a room (the same room used for the TSST procedure but with no people or camera present).

### 2.5. Mental Rotation Task

This task was a computerized version of the one originally developed by Shepard and Metzler [28]. Participants were seated in front of a computer screen, and on each trial (4 training trials and 20 test trials), there were five 3D stimuli presented on the screen: One was the target stimuli. Two of the nontarget stimuli were identical to the target, and another two were almost identical. All of the four nontarget stimuli were rotated in space with respect to the target stimulus. The participants were instructed to decide which of the nontarget stimuli were identical to the target stimulus by mentally rotating them. Each trial was displayed for 30 s and was separated from the next one by a 5 s rest period during which a white screen was displayed. On each test trial, a score of 1 or 0 was given, and the scores of 20 trials were then summed, yielding a total score of 0–20 for each participant.

### 2.6. Statistical Analyses

Differences between groups (men, OC, and luteal) in baseline concentrations of cortisol, sAA, testosterone, estradiol, and progesterone were examined using one-way analyses of variance (ANOVAs) followed by Bonferroni post hoc tests.

To examine the effects of stress on HPA-A and SNS, we conducted a repeated-measures ANOVA, with hormonal status group (men, OC, and luteal), stress exposure (stress, control), and time (T1, T2, T3, T4) being the independent variables and either cortisol (for responders) or sAA (for the whole sample) being dependent variables.

To test the main aim addressing the impact of stress exposure on mental rotation performance, we performed a three-way mixed analysis of variance (ANOVA), with the independent variables being performance (before and after), hormonal status group (men, OC, and luteal), and stress exposure (stress vs. control), and the dependent variable being mental rotation performance.

Because cortisol, sAA, and sex hormone levels were not normally distributed, they were subject to a log 10 transformation. Due to the large variability among participants in their cortisol reactivity to psychosocial stress, we divided the sample into responders (*n* = 20; 9 men, 10 OC, and 12 luteal) and nonresponders (*n* = 31; 9 men, 6 OC, and 5 luteal) according to Hidalgo et al. [29]. Responders were those participants who had an increase in salivary cortisol concentration from baseline levels (−40 min) to the third cortisol measurement (+10 min) after the TSST (the distribution of responders among hormonal status groups did not differ significantly: *χ^2^* (2) = 1.58, *p* > 0.05). For sAA, all participants demonstrated elevated levels from T1 to T2 (no significant difference was found in the increase levels between hormonal status groups: F(2,43) = 0.19, *p* = 0.83). An elaborated description of the findings regarding stress-induced differences in HPA-A and sympathetic responses among hormonal status group has been previously reported [19]. To examine the involvement of stress markers and sex hormones in modulating the effects of stress on mental rotation, we conducted a two-way mixed ANOVA with time and group being the independent variables, performance on the scores on the mental rotation task being the dependent variable, and sex hormones and stress biomarker reactivity as covariates. For this analysis, cortisol and sAA reactivity were calculated as the increase from baseline to peak (T3 (ΔC) and T2 (ΔsAA), respectively (for meta analyses, see [27]). We used Greenhouse–Geisser corrections when the requirement of sphericity in the ANOVA tests for repeated measures was violated. In addition, effect sizes were calculated for every ANOVA test (partial eta squared; *η*^2^_p_). In case of post hoc analyses, data were corrected with Bonferroni’s test for multiple comparisons.

Finally, to further examine the relation between the HPA-A/SNS and the hypothalamic–pituitary–gonadal axis (HPG-A) cross-talk and stress-induced alterations in mental rotation performance, we used a model of interaction analysis. Moderated regression analyses were conducted using mean-centered predictors to calculate interaction terms. The interaction terms were inserted as predictors in the second step of each analysis to predict mental rotation performance (calculated as the difference between before and after stress exposure in mental rotation scores). Furthermore, Pearson’s correlations were calculated to determine the association between sex hormones, cortisol, and sAA reactivity and mental rotation performance (the difference between before and after stress exposure in mental rotation) in each group.

## 3. Results

Table 1 presents the mean baseline concentrations of cortisol, sAA, testosterone, estradiol, and progesterone for each hormonal status group—men, OC women, and LP women.

### 3.1. Stress Response

A significant time (T1, T2, T3, T4) × stress (stress/control) interaction (F(3,282) = 13.20, *p* < 0.001; *η*^2^_p_ = 0.12) was found regarding the levels of cortisol, with post hoc analysis revealing that cortisol level at T3 was higher than cortisol measures at the other three time points in the TSST group but not in the control group. Similarly, a significant time (T1, T2, T3, T4) × stress (stress/control) interaction (F(3,270) = 17.24, *p* < 0.001; *η*^2^_p_ = 0.16) was found regarding the levels of sAA, with post hoc analysis revealing that sAA level at T2 was higher than sAA measures at the other three time points in the TSST group but not in the control group. Finally, there were no significant interactions between stress (stress/control) and hormonal status group (men, OC, LP) with either sAA or cortisol being the dependent variable.

#### 3.1.1. Mental Rotation

The differences between the hormonal status groups in mental rotation performance before and after exposure to either the stress procedure or a nonstress control condition are depicted in Table 2. In a three-way repeated-measures ANOVA with mental rotation as the dependent variable before and after the TSST, no significant main effect was found for the stress group (F(1,100) = 0.41, *p* = 0.525; *η*^2^_p_ = 0.00). However, a significant main effect for the hormonal status group was found (F(1,100) = 4.79, *p* < 0.01; *η*^2^_p_ = 0.09), with post hoc analysis revealing that men outperformed women in both the LP and OC groups (*p* < 0.001). A significant main effect for time was also found (F(1,100) = 8.99, *p* < 0.005; *η*^2^_p_ = 0.08), with performance improving after the TSST procedure (*p* < 0.001). The following interactions were nonsignificant: hormonal status group × time interaction (F(2,100) = 0.10, *p* = 0.902; *η*^2^_p_ = 0.00); hormonal status group × stress interaction (F(2,100) = 2.36, *p* = 0.10; *η*^2^_p_ = 0.05), and hormonal status group × time × (F(2,100) = 1.36, *p* = 0.262; *η*^2^_p_ = 0.03). However, there was a significant stress × time interaction (F(1,100) = 4.40, *p* = 0.038; *η*^2^_p_ = 0.04; see Table 2). Decomposing the interactions revealed that in the control group, no significant difference in performance was found before and after exposure to the nonstress condition (*p* = 0.546). However, for the stress group, performance on the mental rotation improved significantly after the TSST (*p* < 0.001).

#### 3.1.2. Cortisol, sAA, Sex Hormones, and Mental Rotation

To test the relation between the HPA-A and HPG-A cross-talk and mental rotation performance, we first performed a two-way repeated-measures ANOVA with the dependent variable being mental rotation before and after stress and the group as a between-subject factor. A significant main effect was found for stress (F(1,49) = 14.91, *p* < 0.001; *η*^2^_p_ = 0.23), with performance on mental rotation increasing after stress exposure. However, in a reanalysis controlling sex hormones and ΔC, the main effect for stress disappeared (F(1,39) = 0.27, *p* = 0.609; *η*^2^_p_ = 0.01). Furthermore, the main effect for stress disappeared when sex hormones and ΔsAA were controlled (F(1,29) = 0.95, *p* = 0.337; *η*^2^_p_ = 0.03). Hierarchical regressions were conducted to further examine the patterns of joint modulation that sex hormones, cortisol, and sAA reactivity have on mental rotation performance. No statistically significant interactions emerged. Next, Pearson’s correlations were conducted to examine the role of sex hormones and cortisol and sAA reactivity in mental rotation performance. The analysis revealed that estrogen was negatively correlated with the difference in mental rotation performance before and after stress exposure (*r* = −0.529, *p* < 0.02). That is, lower estrogen levels were associated with facilitation in mental rotation performance after the TSST among responders. Furthermore, among men, ΔC was positively correlated with the difference in mental rotation performance before and after stress exposure (*r* = 0.779, *p* < 0.02). That is, higher increase in cortisol levels was associated with facilitation in mental rotation performance after the TSST among male responders.

## 4. Discussion

The current study aimed at examining the possible role of the major neurobiological stress systems (SNS and HPA-A) and sex hormones in the effects of psychosocial stress on mental rotation performance. We hypothesized that the stress-induced modulation of mental rotation performance would be affected by the levels of particular sex hormones, reactive cortisol, and alpha-amylase.

### 4.1. Sex Differences in Baseline Levels of Sex Hormones, Stress Markers and Mental Rotation

As previously reported [30,31], men outperformed women in mental rotation performance. There were no differences between the hormonal status groups (or between men and women of both groups) in the levels of cortisol and sAA prior to exposure to stress. In relation to baseline levels of sex hormones, men had higher testosterone levels than both groups of women, and women had higher basal levels of progesterone and estradiol as compared to men. In line with the hypothesized suppression of sex hormones by oral contraceptives [32], the levels of pre-stress progesterone and estrogen were higher in LP women than in OC women.

### 4.2. Effects of Psychosocial Stress on Mental Rotation Performance

In situations such as driving, individuals under stressful conditions are required to make critical decisions that rely on visuospatial performance. The findings of the current study demonstrate psychosocial stress enhances mental rotation performance. The effects of stress on visuospatial performance were only scarcely examined to date. Specifically, few previous studies have examined the influence of stress on spatial navigation, which at least partially depend on visuospatial abilities, producing mixed results. Performance in a virtual Morris maze task was improved among men following cold pressure stress in one study, but in another study, exposure to the TSST did not affect performance on a similar task among men and impaired it among women [21]. In another study [5], a mild stressor (star mirror tracing task) administered prior to a perspective taking task that required mental rotation capabilities slowed the response of the participants but did not affect the accuracy of their judgment. However, the stressor did not cause any increase in salivary cortisol and stress-induced increase in SNS activation returned to baseline levels prior to the beginning of the visuospatial task. By contrast, the current study employed an effective psychosocial stressor, and a heightened physiological stress response (particularly of the SNS) was evident during performance of the mental rotation task.

### 4.3. Involvement of Neurobiological Stress Systems and Sex Hormones in Stress-Induced Enhancement of Mental Rotation Performance

The results of the current study support the notion that both sex hormones and neurobiological stress mechanisms play a role in the stress-induced improvement in mental rotation performance. Specifically, baseline levels of all sex hormones did not correlate with the pre-stress performance on the mental rotation task, and testosterone and progesterone were not correlated with the stress-induced facilitation in performance. However, baseline estradiol levels were negatively correlated with the level of stress-induced facilitation of mental rotation performance. Thus, estradiol may play a particularly important role in the effects of stress on visuospatial performance.

Notably, this pattern of results, in which the effects of stress are related to estradiol levels but not to levels of progesterone, has been previously reported. For example, Sita and Miller [33] demonstrated that in LP women, cardiovascular activity in response to stressors was negatively associated with levels of estradiol but not of progesterone. Moreover, in a study by Antov and Stockhorst [34], women in the midcycle phase (high estradiol, low progesterone) demonstrated better extinction recall when fear acquisition had been preceded by stress, while in women in the early follicular phase (low estradiol, low progesterone), the inverse was true. Together with the results of the current study, these findings imply that estrogen and progesterone may differ substantially in their involvement in the physiologic and cognitive responses to stressors.

In the current study, the stress-induced increase in the levels of sAA was not correlated with the level of stress-induced facilitation of mental rotation performance. However, among the male participants, there was a positive correlation between the stress-induced increase in the levels of cortisol and the degree of stress-induced facilitation of mental rotation performance. This finding is consistent with a recent report of a correlation between baseline (i.e., not stress-induced) cortisol levels and visuospatial performance, though the effect was observed in both men and women [10]. Interestingly, Hakamata et al. [10] report that cortisol levels in their study were positively associated with the degree of functional connectivity (measured via fMRI) between the rostral hippocampus and prefrontal cortex as well as the lateral occipital cortex, a region which is known to be involved in visuospatial processing in general, and mental rotation in particular [35]. Further research is needed to examine to what extent these pathways are involved in the enhancing effects of stress on visuospatial performance.

Importantly, the correlation observed in the current study between cortisol increase and improvement in mental rotation performance was observed only among men. This result suggests that the interaction between sex hormones and cortisol secretion plays an important role in the effects of stress on mental rotation performance. Indeed, when sex hormone levels and cortisol increase were statistically controlled for, there was no significate stress-induced increase in mental rotation performance. Moreover, a similar disappearance of the stress-induced improvement in mental performance was also observed when sex hormones were controlled for together with the increase in sAA. Thus, it appears that the main stress mechanisms, the HPA-A and the SNS, interact with sex hormones in mediating the effects of stress on visuospatial performance.

It is important to note that the current study examined the contribution of basal sex hormones to the effects of stress on mental rotation performance. However, given that acute stress is capable of enhancing the secretion of sex hormones [15,36,37], it is conceivable that the stress-induced increase in the levels of sex hormones has contributed to the enhancement of mental rotation performance. Nevertheless, these preliminary findings point to the need for more research on the interactive role of sex hormones and HPA-A and SNS activation as it relates to the effects of stress on visuospatial abilities.

### 4.4. Limitations of the Current Study

The findings of the current study should be taken with caution in light of a few limitations. First, in order to increase the statistical power, we exclusively focused on one menstrual cycle phase (the luteal phase) and, thus, women in the follicular phase were not included in the study. Second, while most previous studies on the effects of stress over cognitive functions were conducted in the afternoon, the current study was conducted in the morning. Given that cortisol levels are particularly high in the morning [24], this difference may account for the relatively low number of cortisol responders (participants demonstrating increased cortisol secretion following the TSST) in the current study. Consequently, the available sample size was modest, which reduced the statistical power. However, conducting the study in the morning may also be viewed as a strength as morning hours are an important part of the daily schedule that has not been well studied in research addressing the effects of exposure to stressors. Third, a single saliva sample was used for assessing the levels of the different sex hormones. This could have caused substantial variability due to the short-time pulsating dynamics of the secretion of these hormones [38]. Last, saliva secretion was stimulated by chewing parafilm, and such stimulation could influence the relative quantity of sAA in the saliva [25]. Yet, as the participants of both the stress group and the control group chewed parafilm to stimulate saliva secretion, this factor could not account for differences between the groups in sAA levels.

## 5. Conclusions

Psychosocial stress enhanced mental rotation performance, and this enhancement was dependent on the joint influence of stress-induced activation of the HPA-A and the SNS and baseline levels of sex hormones. Cortisol reactivity, in particular, was positively associated with the degree of stress-induced enhancement of mental rotation performance among men, and this stress-induced enhancement was negatively associated with the baseline estradiol levels. These results provide preliminary support to the notion that a complex interaction between sex hormones and neuroendocrine stress mechanisms has an important role in the facilitation of visuospatial capabilities under conditions of stress.

## Figures and Tables

**Table 1:** Differences between the different hormonal status groups in baseline levels of sex hormones and stress biomarkers.

	Men (*N* = 37)	OC (*N* = 34)	LP (*N* = 36)	F
	M (SD)	M (SD)	M (SD)	
Cortisol (µg/dL)	0.54 (0.39)	0.58 (0.32)	0.56 (0.39)	0.14
sAA (U/mL)	54.17 (50.99)	43.74 (51.49)	42.77 (40.06)	0.61
Testosterone (pg/mL)	126.13 (59.49)	30.12 (19.87)	43.19 (29.71)	57.85 ***
Estradiol (pg/mL)	2.78 (1.08)	2.57 (0.80)	3.23 (1.13)	3.86 *
Progesterone (pg/mL)	31.59 (30.93)	20.48 (17.82)	136.12 (177.14)	12.15 ***

OC = oral contraceptives; LP = luteal phase; sAA = salivary alpha-amylase. * *p* < 0.05; *** *p* < 0.001.

**Table 2:** Means (SD) for group differences in mental rotation performance before and after exposure to either the stress procedure or a nonstress control condition.

MR Performance	Men (*N* = 21)		OC (*N* = 20)		LP (*N* = 17)	
	Control	Stress	Control	Stress	Control	Stress
	M (SD)	M (SD)	M (SD)	M (SD)	M (SD)	M (SD)
Before stress	12.72 (5.37)	10.61 (5.28)	7.24 (4.86)	9.59 (4.89)	8.58 (4.60)	8.59 (5.71)
After stress	11.72 (5.48)	11.50 (4.77)	7.06 (5.80)	11.06 (5.63)	8.47 (5.25)	10.29 (6.41)

MR: mental rotation; OC: oral contraceptives; LP: luteal phase.
